# Supplementary material for: A Whole Virion Vaccine for COVID-19 Produced via a Novel Inactivation Method and Preliminary Demonstration of Efficacy in an Animal Challenge Model
Source: Vaccines (Basel). 2021 Apr 1;9(4):340. doi: 10.3390/vaccines9040340 (PMC8066708; doi:10.3390/vaccines9040340)
Supplement: Supplementary file 1 [file vaccines-09-00340-s001.pdf]

vaccines

MDPI

Supplementary Materials

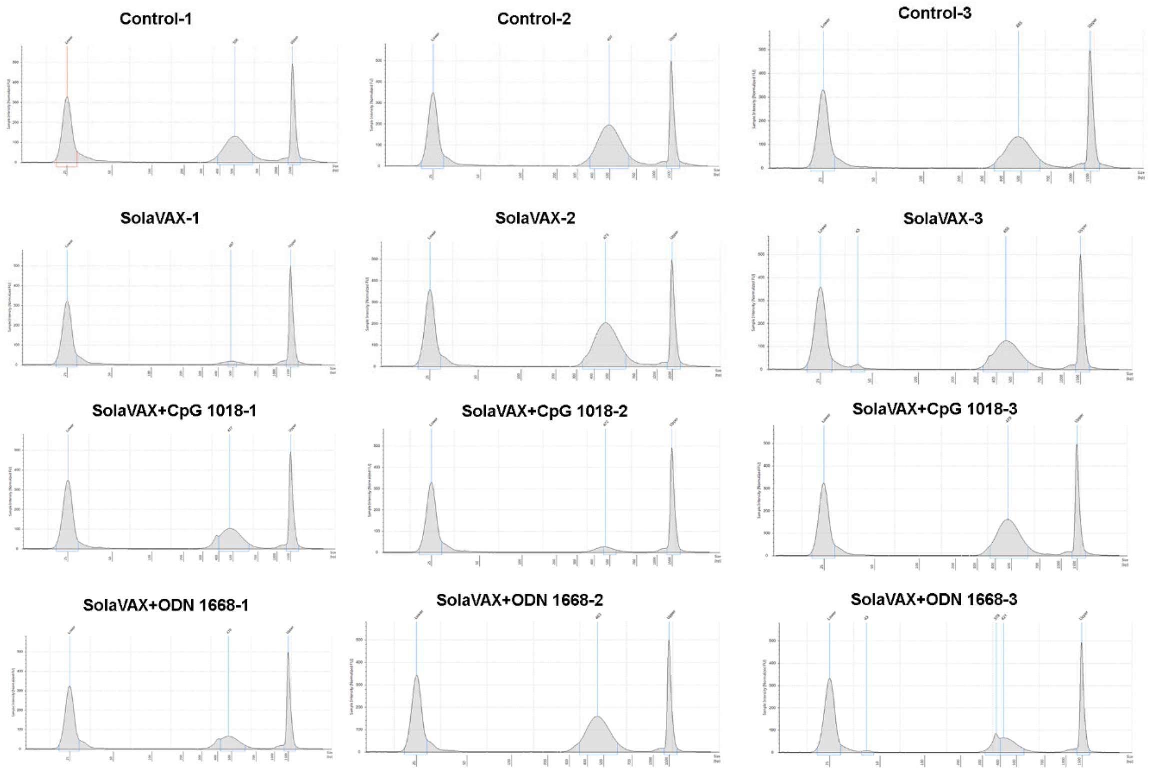

Figure 1. cDNA library traces. cDNA was amplified, library was prepared, and quality and quantity were evaluated via Agilent Tapestation using HS-D1000 screen tapes and reagents. Traces here represent cDNA library after 10-fold dilution.

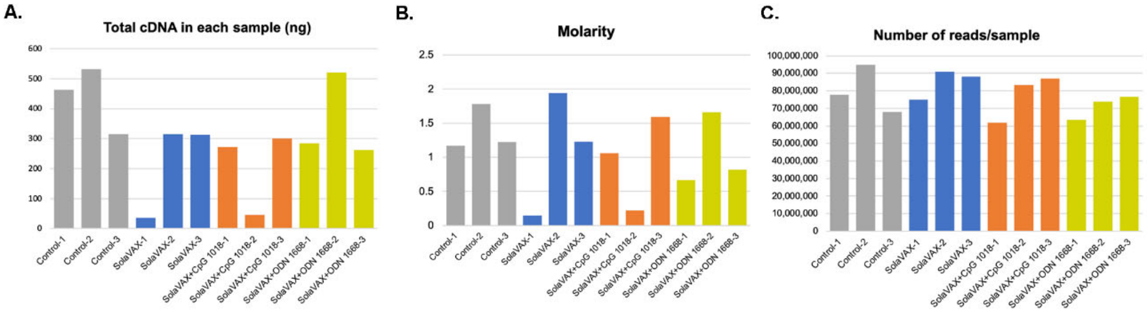

Figure S2. Total cDNA concentration, cDNA library molarity and number of reads for individual samples. Total cDNA in the sample was calculated by taking concentration of cDNA obtained (in pg/μL) between 200-9000 bp. Molarity of the library was

evaluated using region between 250 and 1000 bp. Number of reads were obtained from the combined sequencing run performed in Illumina Next Seq 500.

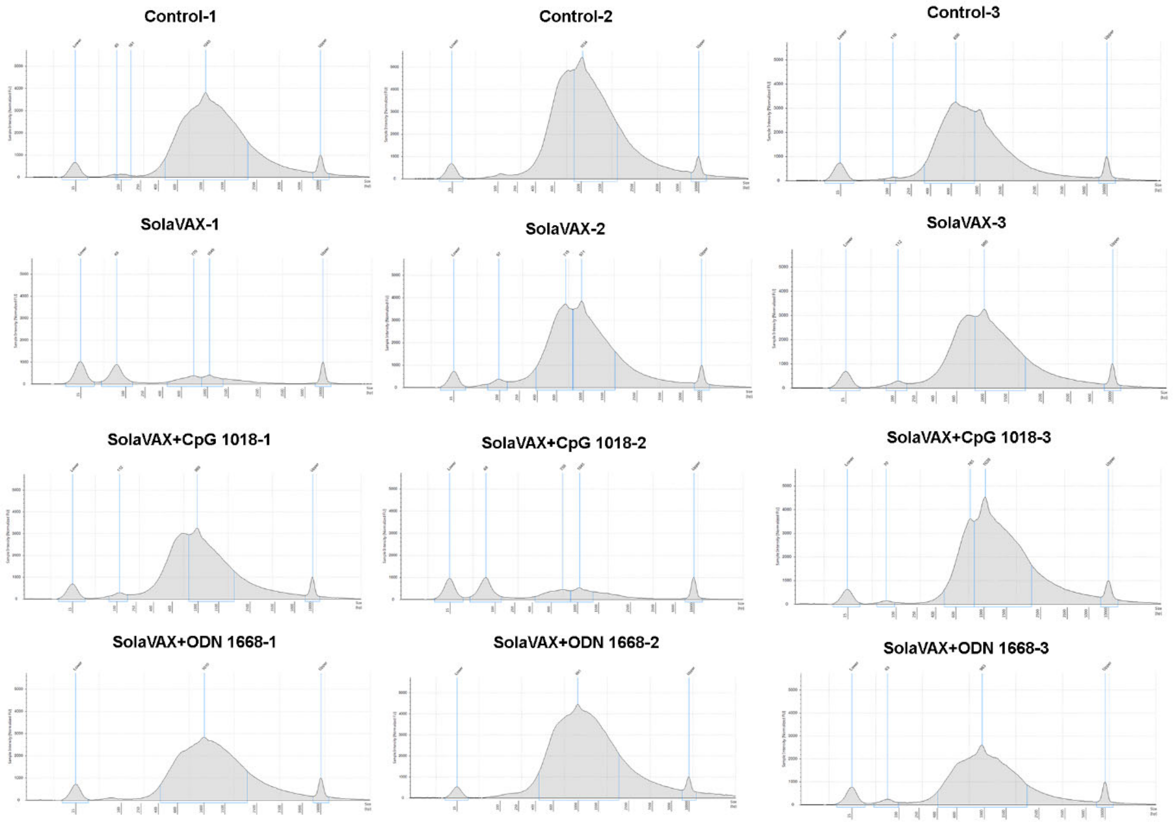

Figure S3. cDNA amplification traces. cDNA was amplified and quality and quantity were evaluated via Agilent Tapestation using HS-D5000 screen tapes and reagents. Traces here represent amplified cDNA after 10-fold dilution.

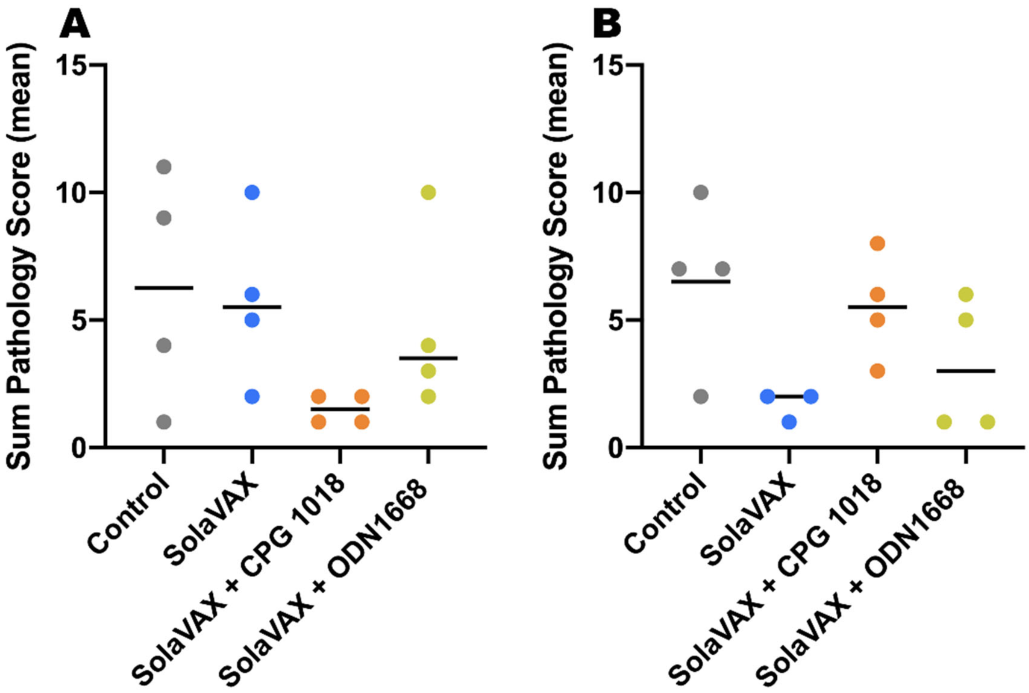

Figure S4. Semiquantitative lung pathology scores from all study groups separated by route of administration. Overall severity of lung pathology was determined by the sum of severity scores for four pathological features with 12 being the maximum assigned sum of severity scores. Data are shown for all groups, separated by intramuscular **(A)** and subcutaneous **(B)** routes of immunization. Data points represent sum scores of individual animals with the bar representing the mean.

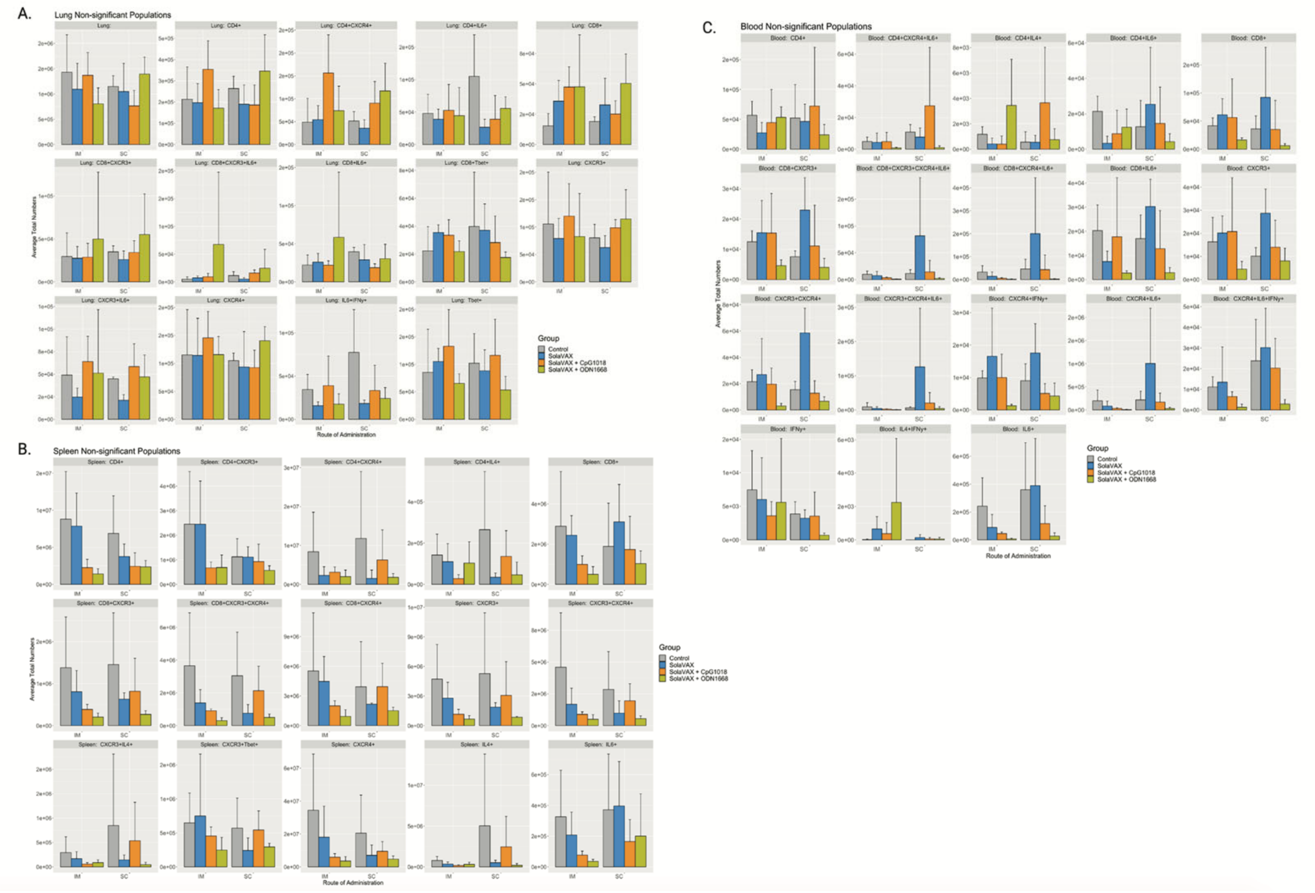

Figure S5. Statistically non-significant flow cytometry populations within intramuscular and subcutaneously vaccinated groups. The bar plots show the statistically non-significant populations for the lung **(A)**, spleen **(B)**, and blood **(C)**. The y-axis displays the average total numbers of cells for the eight groups. The population names at the top of the plots indicate the positive markers in the population: the population is negative for all other markers in the panel.

Table 1. Flow cytometry panel: Flow cytometry panel to study the Th1 and Th2 immune responses in non-vaccinated and SolaVAX vaccinated hamsters. Antibodies were selected based on previous studies and by percent similarity between hamsters to either mouse/rat based on availability of the antibodies.

Antibody	Clone	fluorophore	Concentration of antibody used	Company	Catalogue	References
CD4	GK1.5	Pacific blue	1 µg/mL	Biolegend	100428	[42]
CD8	341	FITC	2 µg/mL	BD Biosciences	554973	[43,44]
IFN-γ	XMG1.2	BV785	1 µg/mL	Biolegend	505838	[45]
IL-10	JES5-16E3	BV421	1 µg/mL	Biolegend	505022	[45]
Gata-3	16E10A23	PE	0.5 µg/mL	Biolegend	653804	[46]
Tbet	4B10	BV711	0.5 µg/mL	Biolegend	644820	[46]
IL-4	11-B11	APC	1 µg/mL	Biolegend	504106	[45]
TNFa	MP6-XT22	PE-Dazzle 594	0.5 µg/mL	Biolegend	506346	Based on percent similarity of protein sequence, hamster and mouse: 82%
IL-6	MP5-20F3	Percp efluor 710	1 µg/mL	Thermo Scientific	46-7061-82	Based on percent similarity of protein sequence, hamster and mouse: 73%
CXCR3	173	APC-Fire 750	1 µg/mL	Biolegend	126540	Based on percent similarity of protein sequence, hamster and mouse: 91.1%
CXCR4	2B11	BV650	2 µg/mL	BD Biosciences	740526	Based on percent similarity of protein sequence, hamster and mouse: 82%
Zombie NIR		Live/dead	1:2000 dilution	Biolegend	423106	Based on percent similarity of protein sequence, hamster and mouse: 93.2%

Table 2. Consensus-changing mutations in the SARS-CoV-2 isolate used for hamster infection studies relative to the USA-WA1 sequence (accession MN985325.1).

Position in genome (nt)[1]	Gene	Nucleotide substitution	Amino acid substitution	Variant frequency[2]
13845	nsp12	U → G	D135E	0.86
22205	S	G → C	D215H	0.90
23616	S	G → A	R685H	0.87
26542	M	C → U	T7I	0.93
28853	N	U → A	S194T	0.94

1. Coordinates relative to MN985325.1
2. Fraction of reads with alternate base

Table 3. Markers for Identification of different cell types. By using principal component analysis, 17 clusters were generated by using Seurat pipeline. These clusters were classified into different cell types based on specific markers cited in literatures.

Markers	Cell type	References
Marco, CD86, CD274, NLRP3, IL-1B	Inflammatory Macrophages	[26]
CD14, Saa3, THBS1, CCL8	Inflammatory monocytes	[26,27]
Marco, CD80, FABP5	Macrophages	[26]
ELANE, NET1, S100A6	Neutrophils	[37]
S100A8, S100A9, CD14	Monocytes	[36]
TCF4, FSCN1, CD83	plasmacytoid dendritic cells	[38,39]
CD3D, CD8A, GZMA, GZMB, NKG7, CD44	Cytotoxic CD8 T cells	[38–40]
CD3D, CD8A, GZMA, NKG7, CD44, XCL	Cytotoxic CD8 T cells, XCL^{hi}	[38–40]
CD3D, CD4, CD44, CD62L, CD38	Central memory CD4+ T cells	[38,39]
CD3D, CD4, CD44, TNFRSF4	Activated CD4+ T cells	[38]
CD3D, GZMA, GZMK, *TNFRSF*	NK cells	[38,40]
CD79B, CD74, H2-Aa, IGJ, IGHM	B cells IGJ^{hi}	[38,41]
CD79B, CD74, H2-Aa	B cells	[38,41]
SFTPC, SFTPB	Type –II alveolar cells	[38]
SFTPC, SOX4	Epithelial progenitor cells	[38]

Reference

36. Mould , K.J.; Jackson, N.D.; Henson, P.M.; Seibold, M.; Janssen, W.J. Single Cell RNA Sequencing Identifies Unique Inflammatory Airspace Macrophage Subsets. JCI Insight 2019, 4, e126556, doi:10.1172/jci.insight.126556.
37. Villani, A.C.; Satija, R.; Reynolds, G.; Sarkizova, S.; Shekhar, K.; Fletcher, J.; Griesbeck, M.; Butler, A.; Zheng, S.; Lazo, S.; et al. Single-cell RNA-seq Reveals New Types of Human Blood Dendritic Cells, Monocytes, and Progenitors. Science 2017, 356, eaaah4573, doi:10.1126/science.aah4573.
38. Zhu, Y.P.; Padgett, L.; Dinh, H.Q.; Marcovecchio, P.; Blatchley, A.; Wu, R.; Ehinger, E.; Kim, C.; Mikulski, Z.; Seumois, G.; et al. Identification of an Early Unipotent Neutrophil Progenitor with Pro-Tumoral Activity in Mouse and Human Bone Marrow. Cell Rep. 2018, 24, 2329–2341, doi:10.1016/j.celrep.2018.07.097.
39. Lee, J.S.; Park, S.; Jeong, H.W.; Ahn, J.Y.; Choi, S.J.; Lee, H.; Choi, B.; Nam, S.K.; Sa, M.; Kwon, J.S.; et al. Immunophenotyping of COVID-19 and Influenza Highlights the Role of Type I Interferons in Development of Severe COVID-19. Sci. Immunol. 2020, 5, eabd1554, doi:10.1126/sciimmunol.abd1554.
40. Szabo, P.A.; Levitin, H.M.; Miron, M.; Snyder, M.E.; Senda, T.; Yuan, J.; Cheng, Y.L.; Bush, E.C.; Dogra, P.; Thapa, P.; et al. Single-cell Transcriptomics of Human T Cells Reveals Tissue and Activation Signatures in Health and Disease. Nat. Commun. 2019, 10, 4706, doi:10.1038/s41467-019-12464-3.
41. Milpied, P.; Cervera-Marzal, I.; Mollichella, M.L.; Tesson, B.; Brisou, G.; Traverse-Glehen, A.; Salles, G.; Spinelli, L.; Nadel, B. Human Germinal Center Transcriptional Programs are De-synchronized in B Cell Lymphoma. Nat. Immunol. 2018, 19, 1013–1024, doi:10.1038/s41590-018-0181-4.
42. Hammerbeck, C.D.; Hooper, J.W. T Cells are not Required for Pathogenesis in the Syrian Hamster Model of Hantavirus Pulmonary Syndrome. J. Virol. 2011, 85, 9929–9944, doi:10.1128/JVI.00203-12.
43. Prescott, J.; Safronetz, D.; Haddock, E.; Robertson, S.; Scott, D.; Feldmann, H. The Adaptive Immune Response does not In-fluence Hantavirus Disease or Persistence in the Syrian Hamster. Immunology 2013, 140, 168–178, doi:10.1111/imm.12116.
44. Gao, Q.; Chen, C.; Ji, T.; Wu, P.; Han, Z.; Fang, H.; Li, F.; Liu, Y.; Hu, W.; Gong, D.; et al. A Systematic Comparison of the Anti-Tumoural Activity and Toxicity of the Three Adv-TKs. PLoS ONE 2014, 9, e94050, doi:10.1371/journal.pone.0094050.

45. Kaewraemruaen, C.; Sermswan, R.W.; Wongratanacheewin, S. Induction of Regulatory T Cells by Opisthorchis viverrini. Parasite Immunol. 2016, 38, 688–697, doi:10.1111/pim.12358.
46. McCann, K.E.; Sinkiewicz, D.M.; Norvelle, A.; Huhman, K.L. De novo Assembly, Annotation, and Characterization of the Whole Brain Transcriptome of Male and Female Syrian Hamsters. Sci. Rep. 2017, 7, 40472, doi:10.1038/srep40472.
